# A Novel Approach for Amplification and Purification of Mouse Oligodendrocyte Progenitor Cells

**DOI:** 10.3389/fncel.2016.00203

**Published:** 2016-08-22

**Authors:** Junlin Yang, Xuejun Cheng, Jiaxi Shen, Binghua Xie, Xiaofeng Zhao, Zunyi Zhang, Qilin Cao, Ying Shen, Mengsheng Qiu

**Affiliations:** ^1^Zhejiang Key Laboratory of Organ Development and Regeneration, The Institute of Developmental and Regenerative Biology, College of Life and Environment Sciences, Hangzhou Normal UniversityHangzhou, China; ^2^The Vivian L Smith Department of Neurosurgery, University of Texas Medical School at Houston, HoustonTX, USA; ^3^Department of Neurobiology, Key Laboratory of Medical Neurobiology of the Ministry of Health, Zhejiang Province Key Laboratory of Neurobiology, Zhejiang University School of MedicineHangzhou, China; ^4^Department of Anatomical Sciences and Neurobiology, University of Louisville, LouisvilleKY, USA

**Keywords:** OPCs, GPCs, coculture, EGF, synergistic effect, PDGFaa, stratified culture

## Abstract

Although transgenic and knockout mice are widely used to study the specification and differentiation of oligodendrocyte precursor cells (OPCs), mouse primary OPCs are difficult to be purified and maintained, and many *in vitro* studies have to resort to rat OPCs as substitutes. In this study, we reported that mouse O4 negative early-stage OPCs can be obtained by culturing cortical tissue blocks, and the simultaneous treatment of OPCs with Platelet Derived Growth Factor-AA (PDGFaa), basic fibroblast growth factor (bFGF), and epidermal growth factor (EGF) is the key for the propagation of mouse OPCs in culture. EGF was found to be a potent mitogen for OPCs and cooperate with PDGFaa to extend cell division and inhibit their differentiation. EGF also collaborates with PDGFaa and bFGF to convert bipolar or tripolar OPCs to more vital fibroblast-like OPCs without compromising their oligodendrocyte differentiation potential. In addition, EGF promoted the survival and proliferation of glial progenitor cells (GPCs) derived from primary OPC cultures, and a mixture of GPCs and OPCs can be obtained and propagated in the presence of EGF, bFGF, and PDGFaa. Once EGF is withdrawn, GPC population decreased sharply and fibroblast-like OPCs changed into typical OPCs morphology, then homogeneous OPCs were obtained subsequently.

## Introduction

In the vertebrate, central nervous system (CNS), oligodendrocytes (OLs) produce myelin sheaths wrapping around axons to facilitate the rapid conduction of nerve impulses and support axonal survival (Marinelli et al., 2016). Oligodendrocyte precursor cells (OPCs) that give rise to OLs were first identified in cultures of postnatal rat optic nerve cells. They are bipotential GPCs, which can differentiate into OLs under defined culture conditions, or into type-2 astrocytes (A2B5^+^/GFAP^+^) in the presence of fetal bovine serum (FBS; Wang et al., 2013). As a result, they were previously termed oligodendrocyte – type 2 astrocyte (O-2A) progenitor cells (Tanner et al., 2011). OPCs were initially characterized in cultures by their antigenic phenotype of A2B5 and their bipolar or tripolar morphology, but more specific molecular markers such as platelet-derived growth factor receptor alpha (PDGFRα) and basic helix-loop-helix transcription factor Olig2 were later identified (Lu et al., 2002; McKinnon et al., 2005). While early-stage OPCs are mostly O4- negative, late-stage OPCs start to acquire O4 antigen with multipolar morphology as they progress along the oligodendroglial lineage (Yang et al., 2011).

Oligodendrocyte precursor cells are widely used as a model system to explore the molecular pathways controlling oligodendrocyte differentiation and axonal myelination *in vitro*, and the pathogenic mechanisms underlying certain demyelinating diseases (Clemente et al., 2013). Establishment of culture conditions for primary OPCs can provide a large number of purified cells for study of development/pathology mechanisms and transplantation-based myelin repair research (Windrem et al., 2004; Cao et al., 2010). To date, the success in isolation and amplification of primary OPCs remains to be restricted to rat tissues. Two different methods have been developed to isolate rat OPCs from brain tissues. One is the cell sorting approach based on cell surface antigen such as immunopanning (Yang et al., 2013) and fluorescence-activated cell sorting (FACS; Sohn et al., 2006), with the cell purity depending on the specific surface antigen. However, many surface antigens of OPCs such as A2B5 and NG2 can be found in other cell types in the CNS (Richardson et al., 2011). The other is the shake method based on the differential adherent properties of glia; however, this method is largely limited to rat tissues due to the difficulty in obtaining stratified culture of OPCs and astrocytes in mouse tissues (Chen et al., 2007). Several studies described methods to obtain mouse OPCs through formation of “oligospheres” from multipotent cortical progenitor cells (Chen et al., 2007; Pedraza et al., 2008), but the scale is limited and their genetic, epigenetic or molecular identity may depart from their *in vivo* counterparts. As mice are widely used in transgenic and knockout studies, it becomes increasingly important to study the molecular or signaling mechanisms underlying the phenotypic chances in oligodendrocyte differentiation or axonal myelination with purified OPC cells *in vitro* (Clemente et al., 2013; White and Krämer-Albers, 2014).

In this study, we developed a new method to obtain stratified cultures of mouse cortical OPCs and astrocytes, and most OPCs separated from the astroglial cell layer by shaking were O4- with bipolar or tripolar morphology. Epidermal growth factor (EGF) was found to be a potent mitogen for OPCs and synergized with Platelet Derived Growth Factor-AA (PDGFaa) to promote cell division and inhibit their differentiation into O4+ cells. More importantly, EGF promotes morphological change of bipolar or tripolar OPCs to fibroblast-like GPCs, maintains the committed oligodendrocyte differentiation potential and functions as a potent mitogen. EGF-dependent tripotential GPCs derived from primary OPC cultures were capable of divisions as well, and provided nourishments for OPCs in the mixed cultures. Upon EGF withdrawal from culture medium, fibroblast-like OPCs reversed to bipolar or tripolar morphology and GPCs reduce gradually, then homogeneous young OPCs were obtained.

## Materials and Methods

### Cortical Tissue Dissociation

Mouse cortices were isolated from postnatal day 1 mouse pups (Chen et al., 2007) in accordance with the NIH Guide for the Care and Use of Laboratory Animals. Cortical tissues were diced into ∼1 mm^3^ pieces in a 60 mm dish with a sterilized razor blade, and the minced tissues were resuspended with freshly prepared D/F20S medium: Dulbecco’s Modified Eagle Medium: Nutrient Mixture F-12 (DMEM/F-12; Gibco, Grand Island, NY, USA) supplemented with 20% v/v FBS (HyClone, Logan, UT, USA) and 1% v/v Penicillin/streptomycin (P/S; Gibco; 5 ml D/F20S medium added for each brain). Tissues were then pipetted up and down until homogenized, transferred into flasks (material from one brain to one T25 flask; Corning) and incubated with 5% CO_2_ at 37°C. Seventy-two hours after plating medium was replaced, most cortical pieces attached onto flasks and some flat cells started to migrate out of tissues. Afterward, medium was replaced every other day.

### OPC Isolation and Culture

With continuous migration of flat cells out of the tissue pieces, some process-bearing cells appeared on the top of bed layer of flat cells. Around the 10th day after plating, mixed glial cultures became confluent. Floating cells were removed by gently rinsing the cell cultures twice with D/F20S medium, and culture flasks were shaken for 15–18 h (37°C, 250 rpm) with tightened caps on an orbital shaker model 420, Orbital Size 1.0 (Thermo Fisher, Waltham, MA, USA). Cell suspension was collected and filtered through Cell Strainer of 40 μm pore size (BD, Franklin Lakes, NJ, USA) to remove small clumps of astrocytes, and then pelleted by centrifugation at 100 × *g* for 5 min at RT. Cells were resuspended in OPC basal media: DMEM/F12 (Gibco) supplemented with 1 × N2 (Gibco), 1 × B27 (Gibco), 1 × P/S (Gibco), and 0.1% w/v BSA (Sigma) plus 10 ng/ml PDGFaa (PeproTech, Rocky Hill, NJ, USA), and then transferred to untreated Petri dishes (Corning) and incubated for 30 min, allowing astrocytes and microglia to attach to the surface, while OPCs remained suspended. Cells in suspension were then collected and counted using a hemocytometer before plated into Poly-D-lysine (PDL; Sigma, 100 μg/mL) and Laminin (LN; Sigma, 20 μg/mL; PDL/LN) coated dishes or plates (Corning) as needed.

### OPC Differentiation

For the directed differentiation of OLs, mouse OPCs were cultured in PDL/LN-coated chamber slides (Millipore, Temecula, CA, USA) with OPC basal media without any growth factor for 3 days, then examined for O4 and MBP antigen expression. For astrocyte differentiation, OPCs were cultured in PDL/LN-coated chamber slides with DMEM/F12 (Gibco) containing 10% v/v FBS (HyClone) and 1 × P/S for 6 days, and then immunostained with A2B5 and GFAP antibody.

### Effects of Growth Factors on OPC Proliferation and Differentiation

For the analysis of cell proliferation and differentiation, 1 × 10^4^ OPCs freshly prepared from the astroglial cell layer were plated to each PDL/LN-coated 24-well before different growth factors or combinations were added. Cell proliferation was analyzed by adding EDU (Life Technologies, Waltham, MA, USA) to a final concentration of 10 μM. Following 24 h of incorporation, cells were fixed in 4% paraformaldehyde at RT for 10 min, and EDU positive cells were detected by Click-iT^®^ EdU Alexa Fluor^®^ 488 Imaging Kit (Life Technologies), followed by immunostaining with O4 or MBP antibody. Double positive cells were counted from three different areas of each well under fluorescence microscopy. The results were expressed as mean values and standard deviation.

### Preparation of Mixed Cultures of OPCs and GPCs

After detached from the bed layer of flat cells, mouse cortical OPCs were suspended in OPC basal media supplemented 10 ng ml^-1^ EGF (PeproTech), bFGF (PeproTech), and PDGFaa (PeproTech) and plated at a density of 1 × 10^3^ cells/cm^2^ into PDL/LN-coated 100 mm dishes. Cells were incubated in 5% CO_2_ at 37°C, and medium was changed every other day. Early-stage OPCs and few GPCs among the primary OPC cultures divided rapidly under the stimulation of EGF + bFGF + PDGFaa. OPCs and GPCs are more firmly attached to dishes and can only detach from dishes by enzymatic treatment instead of by shaking. Following several passages with the aid of trypsinization, the fibroblast-like OPCs and GPCs increased to about 20–40% based on their morphology, and the trypsinization did not appear to reduce cell replication significantly for the first several passages.

### OPC Amplification from Mixed Cultures of OPCs and GPCs

To grow mouse OPCs in large numbers, we plated the mixed culture of OPCs and GPCs at a low density of 1 × 10^3^ cells/cm^2^ on PDL/LN-coated 100 mm dishes, in culture medium supplemented with bFGF (PeproTech) and PDGFaa (PeproTech), and replaced half the medium every other day. In the absence of EGF, GPCs division slowed down, then followed by apoptosis, whereas fibroblast-like OPCs reversed to bipolar or tripolar morphology and continued to proliferate. The mixed cells were split (1:5 ratio) every 5 days for two passages, and high-purity OPCs were obtained.

### Dorsal Root Ganglion Neurons (DRG) Co-culture for Myelination Assays

Similar to OPCs in mice, DRGs were also difficult to prepare, so we have to resort to their rat counterpart as substitutes. DRGs were isolated from E15 Sprague-Dawley rat embryos as previously described (Dadsetan et al., 2009), and cultured for neurite outgrowth as described by Dincman et al. (2012). OPCs/DRGs co-cultures were maintained 8–12 days in OPC basal media supplemented with 30 ng/ml Triiodothyronine (T3; Sigma), and fixed for immunostaining with anti-MBP and anti-neurofilament (axons) antibodies.

### Immunofluorescence Staining Analysis

Immunostaining analysis was performed as previously described (Yang et al., 2009; Dincman et al., 2012). Anti-mouse A2B5 IgM and anti-mouse O4 IgM (50%, v/v) were produced by hybridoma culture. Anti-mouse Olig2 (1:1000), anti-mouse GFAP (1:1000), and anti-rabbit neurofilament (1:1000) were purchased from Millipore. Anti-rat MBP (1:500) was obtained from Abcam, anti-rabbit β-tubulin (1:1000) from Sigma, and anti-rabbit PDGFRα (1:500) from Santa Cruz. The Alexa-488 or Alexa-594 conjugated secondary antibodies were purchased from Invitrogen. The nucleic acid dye 4′,6-diamidino-2-phenylindole (DAPI) was obtained from Roche. All quantitative data are presented as means ± SD. Statistical significance of the difference was evaluated by Student’s *t*-test. *P* < 0.05 was considered statistically significant.

## Results

### Preparation of Mouse Primary OPCs from Cortical Tissues

**FIGURE 1:**
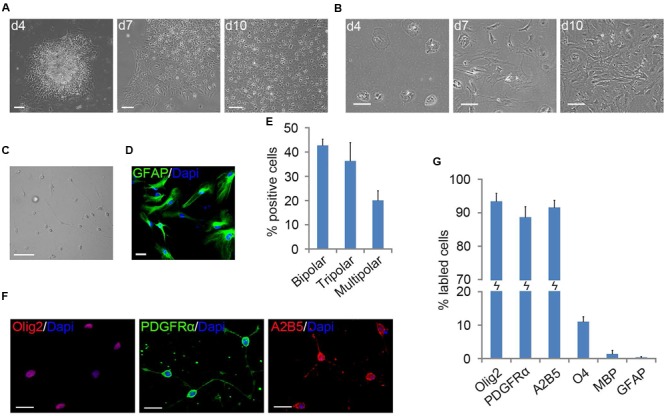
**Primary mouse oligodendrocyte precursor cells (OPCs) derived from neonatal cortical tissue culture. (A)** Flat cells migrated out of the mouse cortical tissues after 4 days of culture to form a bed layer, on top of which cortical OPCs appeared on d7 and become confluent around the 10th day. **(B)** Dissociated mouse cortical cells plating, stratified cultures of OPCs and astrocyte were not formed. **(C)** Morphology of isolated OPCs. **(D)** Flat cells are GFAP+ astrocytes. **(E)** Quantification of bipolar, tripolar and multipolar cells in primary mouse OPCs cultures, *n* = 3. **(F)** OPCs were immunoreactive to Olig2, A2B5, and platelet-derived growth factor receptor alpha (PDGFRα) antibodies. **(G)** Quantification of Olig2, PDGFRα, A2B5, O4, MBP, and GFAP positive cells in isolated primary OPCs cultures, *n* = 5. Statistical analyses are presented as mean ± SD. Scale bars **(A–C)**: 100 μm, **(D,F)**: 50 μm.

When mouse cortical tissues were dissociated with trypsin and plated in PDL/LN-coated flasks with D/F20S medium, stratified cultures of OPCs and astrocytes were not formed as described in rat OPC preparations (**Figure 1B**) (Chen et al., 2007). However, when mouse cortical tissues were diced and plated directly without trypsinization, flat cells migrated out of tissue chunks and formed a bed layer, on top of which small surface cells appeared (**Figure 1A**). The vast majority of small surface cells displayed two or three fine cell processes (**Figure 1A**), characteristic of OPC morphology (Marinelli et al., 2016).

**FIGURE 2:**
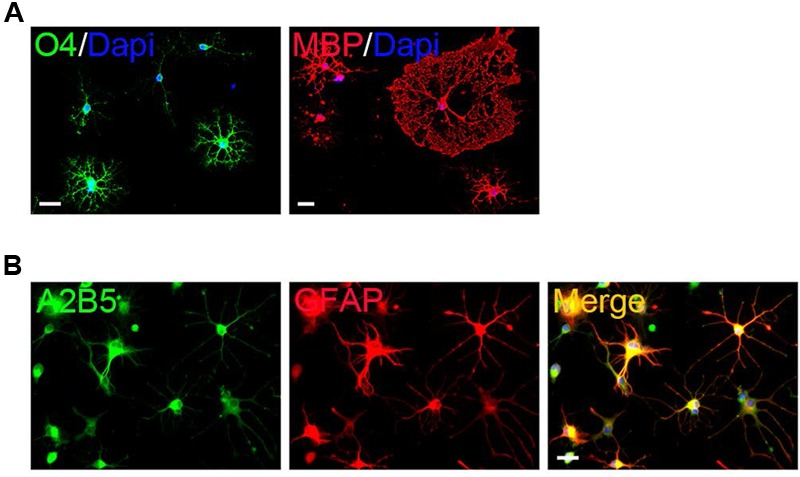
**Differentiation of primary mouse cortical OPCs. (A)** OPCs acquired O4 antigen and differentiated into MBP+ mature when cultured in the differentiation medium. **(B)** OPCs differentiated into A2B5+/GFAP+ type-2 astrocytes when cultured in fetal bovine serum (FBS)-containing media. Statistical analyses are presented as mean ± SD. Scale bars: 50 μm.

The surface OPCs can be physically separated by shake force from the bed layer of GFAP+flat cells (**Figure 1D**). After replated onto the PDL/LN-coated dishes in OPC basal media plus 10 ng/ml PDGFaa, OPCs displayed typical morphological features with fine processes (bipolar 42.75 ± 2.54%, tripolar 36.34 ± 7.54%, multipolar 20.09 ± 3.91%; **Figures 1C,E**). Immunofluorescent staining revealed a high percentage of these cells immunoreactive to A2B5, PDGFRα, and Olig2 (96.74 ± 5.77%, 95 ± 7.4%, and 93.08 ± 6.69%, respectively; **Figures 1F,G**), and less than 0.5% of them were GFAP+ (Supplementary Figure S1C; **Figure 1G**), indicating that the vast majority of them possessed the antigenic properties of OPCs (Dincman et al., 2012; Marinelli et al., 2016). However, a small percentage of OPCs expressed O4 antigen (11.05 ± 1.46%; Supplementary Figure S1A; **Figure 1G**) or mature OL marker MBP (∼1%; Nicolay et al., 2007) with multipolar morphology (Supplementary Figure S1B; **Figure 1G**). Neurofilament+ neurons were not detected in the culture (Supplementary Figure S1D) (Shaw et al., 1981). When OPCs were cultured in basal media without PDGFaa for 3 days, they appeared to differentiate into mature OLs rapidly, and O4 expression almost reached the peak around day 3, however, MBP expression is slower than O4 for about 1 day (**Figure 2A**; Supplementary Figure S2). In contrast, when OPCs were cultured in the DMEM/F12 medium supplemented with 10% FBS, the vast majority of them differentiated into A2B5+/GFAP+ type-2 astrocytes by d6 (**Figure 2B**; Supplementary Figure S2). Together, these results indicated that mouse OPCs prepared from cortical tissues displayed typical O-2A antigenic characteristics and differentiation potentials (Dincman et al., 2012).

### EGF Cooperates with PDGFaa to Promote Extended Division and Self-Renewal of Mouse Cortical OPCs

Although PDGFaa potently promotes proliferation of rat OPCs (Noble et al., 1988; Raff et al., 1988; Hart et al., 1989; Gard and Pfeiffer, 1993; Kleinsimlinghaus et al., 2013), it could not maintain the self-renewal and proliferation of mouse OPCs for a long time period even in the presence of bFGF or NT-3 (Supplementary Figure S3), suggesting that other important factors are also required for the sustained proliferation of mouse OPCs. The previous observation that EGFR overexpression expanded OPC pool *in vivo* (Aguirre et al., 2007) prompted us to examine the effect of EGF on the proliferation and maintenance of mouse cortical OPCs. Following 1-day EGF treatment (10 ng/ml), more than half of cells (52.49 ± 2.06%) were found EDU-positive in the cultures, which is comparable to cultures treated with PDGFaa (56.03 ± 3.91%). However, the percentage of EDU-positive cells in EGF-treated cultures decreased quickly thereafter, followed by an increase in MBP+ cells, indicating that EGF alone can stimulate the proliferation of mouse cortical OPCs, but can not prevent their differentiation. We next investigated whether EGF could work synergistically with PDGFaa to promote OPC proliferation and repress differentiation. When mouse cortical OPCs were exposed to PDGFaa alone or EGF + PDGFaa for 4 days, EGF was found to enhance the response of mouse cortical OPCs to PDGFaa to divide (**Table 1**). The cultures exposed to EGF + PDGFaa contained a higher percentage of EDU-positive cells than that treated with PDGFaa alone (*P* < 0.05). EGF also synergized with PDGFaa to inhibit expression of O4 antigen in OPCs, and the percentage of O4+ cells in EGF + PDGFaa treated cultures was reduced by half compared with that exposed to PDGFaa only. At the same time, EGF cooperated with PDGFaa to inhibit OPC differentiation into mature OLs, as the percentage of MBP+ cells in the presence of EGF + PDGFaa was about 4%, as compared to 10.7% for PDGFaa treatment (*P* < 0.05).

**Table 1:** Effects of Platelet Derived Growth Factor-AA (PDGFaa), basic fibroblast growth factor (bFGF), or/and epidermal growth factor (EGF) on oligodendrocyte precursor cell (OPC) proliferation and differentiation *in vitro*.

Condition	EDU positive cells (%)	O4 positive cells (%)	MBP positive cells (%)
PDGFaa	33.12 ± 4.44	56.64 ± 8.19	10.74 ± 3.19
PDGFaa + bFGF	38.70 ± 3.28	25.70 ± 6.88	4.13 ± 1.21
PDGFaa + EGF	37.95 ± 2.41	26.32 ± 5.44	4.01 ± 0.79
PDGFaa + bFGF + EGF	49.89 ± 5.90	15.78 ± 4.54	1.44 ± 0.26

*Data are presented as mean ± SD, *n* = 3.*

Basic fibroblast growth factor was previously reported to amplify the effect of PDGFaa in promoting DNA synthesis and inhibiting differentiation of rat OPCs (Tang et al., 2000). We found that bFGF and EGF had similar effects in synergizing with PDGFaa to promote mouse OPC proliferation and inhibit their differentiation in parallel experiments. More importantly, EGF, bFGF, and PDGFaa had additive effects in enhancing cell proliferation and reducing differentiation (**Table 1**), suggesting that the proliferation and differentiation of mouse cortical OPCs are regulated by multiple signaling pathways.

### A Mixture of OPCs and GPCs Were Induced by Stimulation of EGF + bFGF + PDGFaa

**FIGURE 3:**
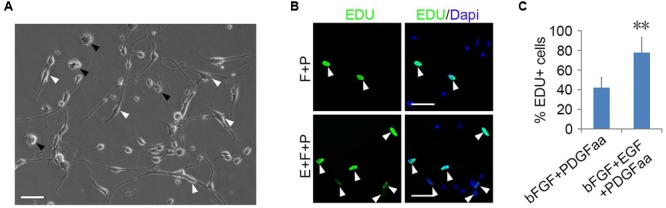
**Epidermal growth factor (EGF) + basic fibroblast growth factor (bFGF) + Platelet Derived Growth Factor-AA (PDGFaa) promoted the proliferation of mixed culture of OPCs and glial progenitor cells (GPCs). (A)** A mixed cultures of GPCs (white arrow) and early-stage OPCs derived from EGF + bFGF + PDGFaa stimulation, while late-stage multipolar OPCs gradually died (black arrow). **(B)** EGF has strong synergistic effect with bFGF + PDGFaa to stimulate division of the mixed culture of OPCs and GPCs. **(C)** The percentage of EDU+ cells in bFGF + PDGFaa and EGF + bFGF + PDGFaa treated mixed culture of OPCs and GPCs, *n* = 6. Abbreviation: F+P, PDGFaa + bFGF; E+F+P, EGF + bFGF + PDGFaa. Statistical analyses are presented as mean ± SD, *n* = 3. ^∗∗^*P* < 0.01, Scale bars **(A)** 100 μm, **(B)** 50 μm.

**FIGURE 4:**
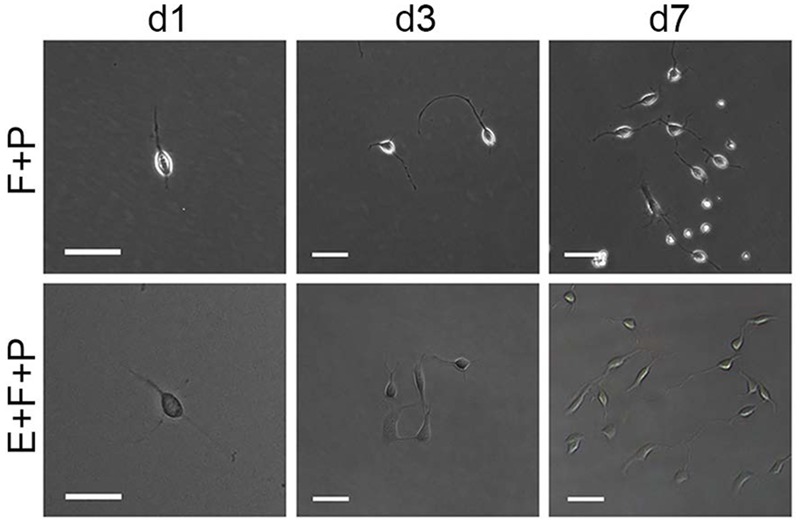
**Effects of EGF on the morphology and proliferation of single bipolar OPCs in culture.** Abbreviation: F+P, PDGFaa + bFGF; E+F+P, EGF + bFGF + PDGFaa. Scale bars: 60 μm.

Although bFGF + PDGFaa and EGF + PDGFaa promoted extended division and inhibited differentiation of mouse cortical OPCs as described above, primary mouse OPCs can only be maintained *in vitro* for a limited period of time. With time, cell divisions slowed down and ceased, and eventually all OPCs underwent apoptosis. However, when the isolated OPC cells were exposed to these three growth factors simultaneously, the early-stage OPCs with bipolar or tripolar morphology maintained strong proliferation ability, while the late-stage multipolar ones gradually died and then disappeared (**Figure 3A**). Unexpectedly, a few EGF-responsive fibroblast-like cells started to appear in primary OPC cultures (**Figure 3A**) and EGF has strong synergistic effect with bFGF + PDGFaa tostimulate division of the mixed culture of OPCs and GPCs (**Figures 3B,C**). Clonal analysis showed that EGF + bFGF + PDGFaa can change the morphology of OPCs, a mixture of bipolar or tripolar OPCs and fibroblast-like cells can be derived from single OPC cell in the presence of these three factors (**Figure 4**). In contrast, bFGF + PDGFaa kept OPC the typical bipolar or tripolar morphology (**Figure 4**).

**FIGURE 5:**
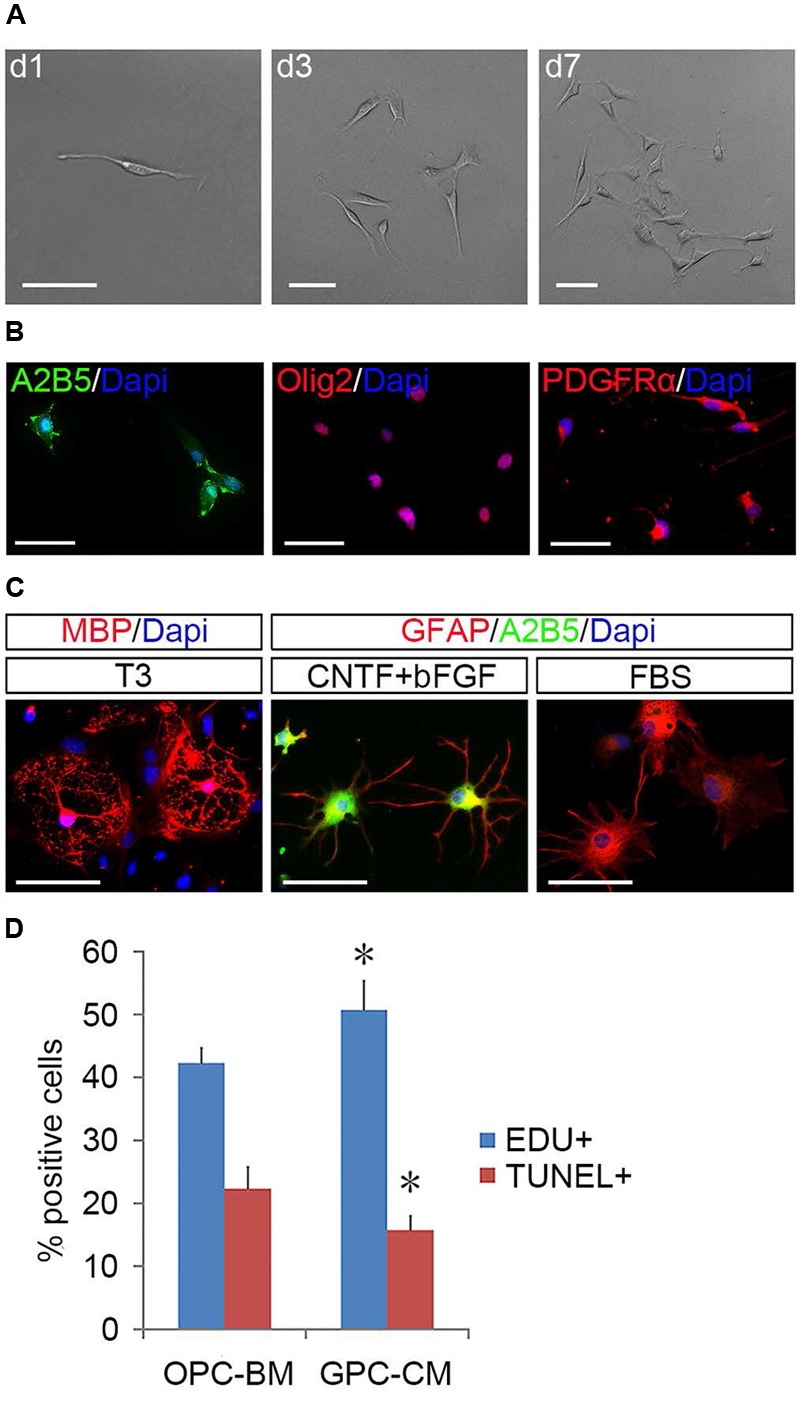
**Identification of GPCs and their nourishing effects on OPCs. (A)** A single fibroblast-like cell derived GPC clone. **(B)** GPCs are immunopositive to Olig2, PDGFRα, and A2B5. **(C)** GPCs differentiate into MBP+ oligodendrocytes in T3 and A2B5+/GFAP+ type-2 and A2B5-/GFAP+ type-1 astrocytes when exposed to CNTF + bFGF and FBS, respectively. **(D)** GPC-CM significantly increased proliferation and reduced apoptosis of OPCs compared to OPC-BM, *n* = 5. Abbreviation: GPC-CM, GPC-conditioned medium; OPC-BM, OPC basal medium. Scale bars **(A)**: 100 μm, **(B,C)**: 50 μm.

To determine the identity of the induced flat cells, we analyzed 50 single clones derived from fibroblast-like cells by immunostaining and directed differentiation. It was found that these fibroblast-like cells were immunoreactive to OPC markers including Olig2, A2B5, and PDGFRα (**Figure 5B**), but negative for GFAP, O1, O4, and neurofilament (Supplementary Figure S4). In the presence of FBS, 29 clones produced A2B5+/GFAP+ type-2 astrocytes; in the parallel cultures, T3 promoted them to differentiate into MBP+ OLs. The other 21 clones (**Figure 5A**) mainly yielded A2B5-GFAP+ and A2B5+GFAP+ astrocytes in the presence of FBS and CNTF + bFGF, respectively, and MBP+ OLs were detected in T3 culture (**Figure 5C**), indicating that these clones were less committed to oligodendrocyte lineage and displayed the same developmental potentials as the tripotential GPCs (Gregori et al., 2002; Dadsetan et al., 2009; Haas et al., 2012). Intriguingly, GPC-conditioned medium significantly promoted the proliferation of OPCs and reduced apoptosis of them (**Figure 5D**), suggesting that GPCs may provide some unknown nourishments to OPCs to enhance their proliferation and self-renewal in the mixed cultures.

### OPCs Can Be Amplified from the Mixture of OPCs and GPCs

**FIGURE 6:**
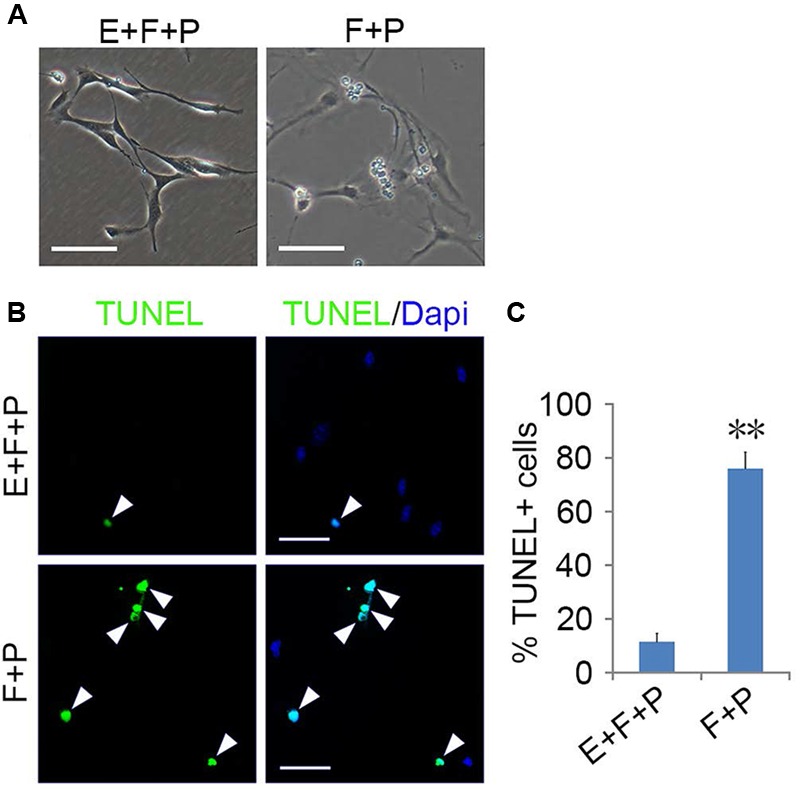
**EGF promoted the survival of GPCs. (A)** Morphology of GPCs treated with EGF + bFGF + PDGFaa and bFGF + PDGFaa. **(B)** GPCs underwent apoptosis in the absence of EGF even when bFGF + PDGFaa were present. **(C)** Quantification of TUNEL+ cells in bFGF + PDGFaa and EGF + bFGF + PDGFaa treated GPCs, *n* = 5. Abbreviation: F+P, PDGFaa + bFGF; E+F+P, EGF + bFGF + PDGFaa. Statistical analyses are presented as mean ± SD. ^∗∗^*P* < 0.01, Scale bars **(A)** 100 μm, **(B)** 50 μm.

**FIGURE 7:**
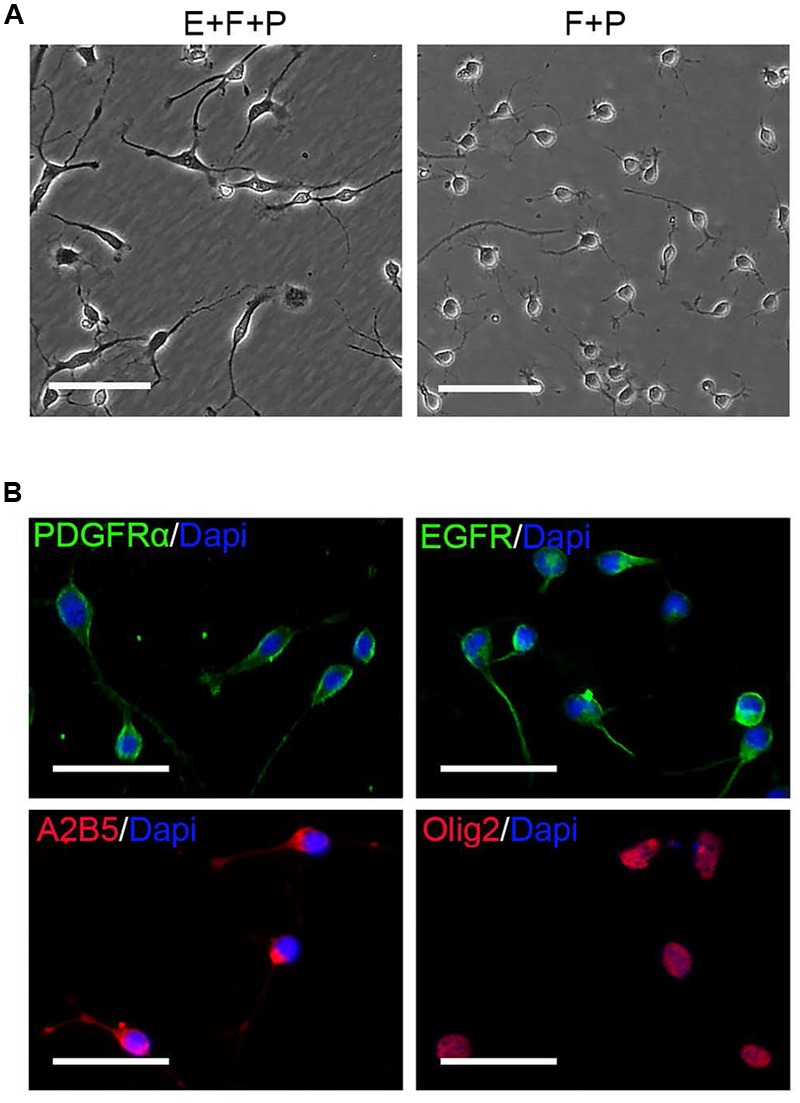
**EGF withdrawal reversed GPCs to OPCs. (A)** Single OPC derived clone treated with EGF + bFGF + PDGFaa (left image) reversed to typical OPC morphology after EGF withdrawal (right image). **(B)** OPCs derived from mixed culture of OPCs and GPCs after EGF withdrawal are immunopositive for PDGFRα, EGFR, A2B5, and Olig2. Abbreviation: F+P, PDGFaa + bFGF; E+F+P, EGF + bFGF + PDGFaa. Scale bars **(A)** 100 μm, **(B)** 50 μm.

EGF signaling promoted the emergence of fibroblast-like GPCs in primary OPC culture; however, these EGF-responsive GPCs were difficult to survive the absence of EGF. When EGF was withdrawn, the division of GPCs slowed down and underwent apoptosis despite the presence of bFGF and PDGFaa (**Figures 6A–C**), resulting in a continuous decline of the fibroblast-like cell population in the mixed culture. Interestingly, fibroblast-like OPCs derived from EGF + bFGF + PDGFaa treatment reversed to bipolar or tripolar morphology after EGF withdrawal (**Figure 7A**). One possible reason for the disappearance of fibroblast-like cells in the mixed cultures following EGF withdrawal could be due to the apoptosis of GPCs and the morphological changes of OPCs.

**FIGURE 8:**
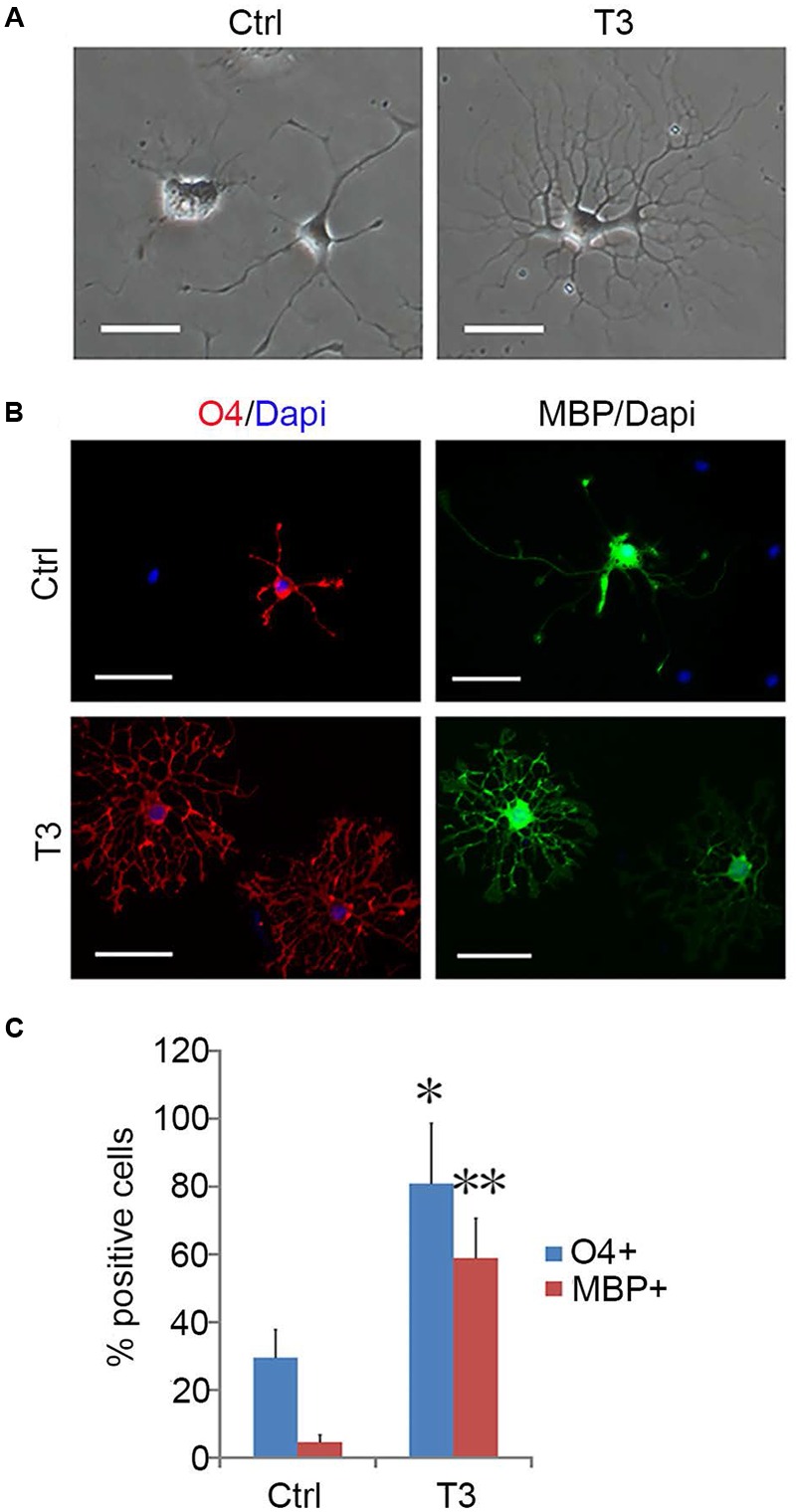
**T3-dependent differentiation of early-stage OPCs. (A)** The morphology of OPCs in the presence or absence of T3 for 3 day. **(B)** T3 induction of O4+ and MBP+ oligodendrocytes. **(C)** Quantification of O4+ and MBP+ cells induced by T3, *n* = 5. Statistical analyses are presented as mean ± SD. ^∗^*P* < 0.05, ^∗∗^*P* < 0.01, Scale bars **(A)** 100 μm, **(B)** 50 μm.

OPCs continued to proliferate for a period of time even when fibroblast-like cells were difficult to find, and they continued to display typical bipolar or tripolar morphology and immunoreactivity to Olig2, A2B5, PDGFRα (**Figure 7B**). O4+ cells were not observed in the culture, indicating that they remained as early-stage OPCs. In addition, these OPCs were more dependent on T3 for oligodendrocyte differentiation than the initially isolated OPCs. When OPCs were cultured in basal media plus T3 for 3 days, multiple and highly branched processes grew out rapidly until typical oligodendrocyte morphology emerged (**Figure 8A**). In the absence of T3, the processes were much shorter or less branched (**Figure 8A**). Immunological analysis confirmed the higher percentage of O4+ and MBP+ cells (80.89 ± 17.81% and 58.93 ± 11.69%, respectively) in T3 differentiation culture, as compared to the culture without T3 (29.59 ± 8.24% and 4.65 ± 2.07%, respectively; **Figures 8B,C**).

**FIGURE 9:**
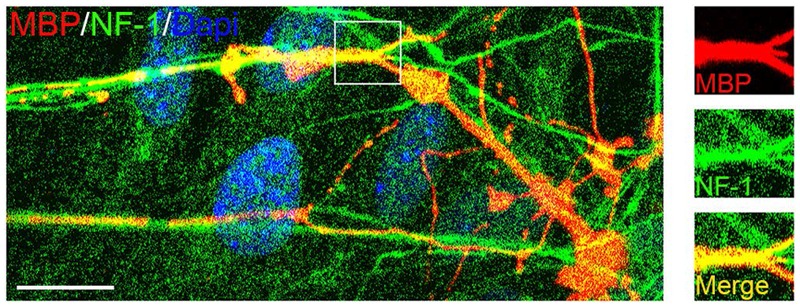
**Confocal image of myelinated axons in OPC/DRG co-culture.** Purified OPCs matured into MBP+ oligodendrocytes (OLs) in the co-culture, and aligned with axons marked by neurofilament (NF1) staining. Scale bars: 10 μm.

To test whether mouse OPCs derived from the mixed culture retained their capacity to myelinate axons, we co-cultured them with embryonic DRGs (Chan et al., 2004) for 2 weeks. Double immunostaining experiments demonstrated that these OPCs gave rise to MBP+ OLs, which formed myelin sheaths around the neurofilament+ axons (**Figure 9**). These results indicated that mouse cortical OPCs maintained their biological characteristics after amplification *in vitro*.

## Discussion

### Species Difference in OPC Isolation and Amplification

Purification and culture of mouse primary OPCs have been a technical challenge (Chen et al., 2007), as they cannot be obtained and maintained by the simple dissociation culture of brain tissues used for preparation of rat OPCs. As a result, many studies are forced to use rat OPCs as the alternative sources to complement their genetic studies in transgenic and mutant mouse models (Chew et al., 2011; Liu et al., 2012; Chen et al., 2013; Zhu et al., 2014). The mechanisms underlying the observed species difference in OPC isolation are currently unknown. One possibility is that mouse OPCs are particularly sensitive to enzymatic digestion and fail to survive the treatments. Use of small cortical tissues can avoid the damage from enzyme digestions. Moreover, small tissue blocks may provide OPCs an environment mimicking their *in vivo* niche, and help them to adapt to the culture conditions and maintain self-renewal. In addition, astrocytes that migrate out of cortical tissues could serve as feeder cells for OPCs and provide favorable environment for OPC migration and proliferation. Astrocyte layers may actively secrete some cytokines that promote OPC survival and proliferation. Interestingly, although rat cortical OPCs can be prepared by dissociation culture of nerve cells, the majority of them are O4+ with multipolar morphology (Bansal et al., 1992). It is plausible that cytokines secreted by rat astrocytes may be sufficient to support the survival of rat OPCs, but ineffective to inhibit their differentiation.

### EGF Signaling Promotes the Proliferation of Mouse OPCs

Epidermal growth factor was previously found to be a mitogen for neural stem cells (NSCs; Deleyrolle et al., 2006; Kanakasabai et al., 2012). In this study, we observed EGF responsiveness in committed mouse glial precursors including OPCs and GPCs. Although EGF is effective in stimulating OPC proliferation, it did not inhibit cell differentiation and OPCs gradually differentiated into MBP+ OLs in EGF treatment alone. PDGFaa inhibits OPC differentiation more effectively than EGF, but PDGFaa alone was not sufficient to sustain self-renewal of mouse O4- early-stage OPCs. Interestingly, EGF and PDGFaa exhibited a synergistic effect in preventing the differentiation of O4- OPCs, analogous to the effect of bFGF and PDGFaa in rat OPCs (Tang et al., 2000). Thus, the influence of EGF on OPCs is not restricted to proliferation, it also regulates OPC differentiation by collaborating with other factors. This is in keeping with the recent observation that EGFR signaling promotes oligodendrocyte proliferation after injuries (Scafidi et al., 2014).

### EGF Signaling Promotes GPC Development

In addition, the EGF-dependent proliferation and survival were observed for mouse postnatal cortical GPCs in primary OPC culture, which means EGF signaling plays an important role in GPC development. Certainly, it can not be excluded that the degree of EGF-dependence may be different for GPCs from different regions and developmental stages. Formerly it was a challenge to maintain a homogeneous culture of GPCs for prolonged periods in culture, and immortalization by constitutive expression of oncogene was forced to be adopted for obtaining a clonal cell line of GPC (Wu et al., 2002). We demonstrated that the single cell derived GPC clones can maintain an undifferentiated dividing state for a long time in presence of EGF + bFGF + PDGFaa *in vitro*. To our surprise, GPC conditioned medium contributed to the dividing progenitor state of mouse cortical OPCs, indicating that some unknown cytokines secreted by GPCs are beneficial for the survival and self-renewal of OPCs *in vitro*. Since OPCs can exist *in vivo* throughout life span, similar factors may be secreted by the surrounding cells in the nervous tissue (El Waly et al., 2014). Elucidation of these cytokines shall contribute to our further understanding of molecular mechanisms governing the survival, division, and differentiation of mouse OPCs.

### EGF Signaling Promotes a Juvenile Stage of Mouse OPCs

The cooperation of EGF with bFGF + PDGFaa promoted morphological change of early-stage OPCs into fibroblast-like cells resembling GPCs in shape. These GPC-like OPCs proliferated quickly with a low apoptosis rate, and they seem to represent OPCs at an early or young stage. Despite their morphological similarities to GPCs, they still retained the oligodendrocyte differentiation potential, and were not fully converted to GPCs. Based on its function in maintaining proliferation and self-renewal of NSCs, GPCs, and early-stage OPCs (Deleyrolle et al., 2006; Kanakasabai et al., 2012), it is postulated that EGF plays an important role in stem cell maintenance and oligodendrocyte development. High concentration of EGF maintains the proliferation and self-renewal of stem cells or progenitor cells which possess potential to differentiate into OLs, and low concentration promotes their differentiation (Scafidi et al., 2014).

In summary, we reported a novel approach for the isolation, amplification, and purification of mouse OPCs by utilizing the EGF dependence of GPCs and the nourishing effects of GPCs on OPCs. High purity of OPCs can be obtained by withdrawing EGF from the mixed cultures of GPCs and OPCs. In addition, GPCs/OPCs mixtures can tolerate cryopreservation, and they can be stored after amplification for later use, avoiding repeated preparations of OPCs from central nervous tissues.

## Author Contributions

JY designed, performed experiments, collected, analyzed the data, and contributed to writing the manuscript; XC, JS, BX, and XZ performed experiments; QC, ZZ, and YS analyzed data; MQ designed, supervised the experiments, collected, analyzed, and discussed data, and wrote the manuscript.

## Conflict of Interest Statement

The authors declare that the research was conducted in the absence of any commercial or financial relationships that could be construed as a potential conflict of interest.
