# Supplementary material for: A Novel Approach for Amplification and Purification of Mouse Oligodendrocyte Progenitor Cells
Source: Front Cell Neurosci. 2016 Aug 22;10:203. doi: 10.3389/fncel.2016.00203 (PMC4992724; doi:10.3389/fncel.2016.00203)
Supplement: Supplementary file 1 [file Data_Sheet_1.PDF]

Supplementary Material

A novel approach for amplification and purification of mouse oligodendrocyte progenitor cells

Junlin Yang, Xuejun Cheng, Jiaxi Shen, Binghua Xie, Xiaofeng Zhao, Zunyi Zhang, Qilin Cao, Ying Shen, Mengsheng Qiu*

*** Correspondence:** Mengsheng Qiu, Ph.D., Department of Anatomical Sciences and Neurobiology, University of Louisville, Louisville, KY 40292, USA. Telephone: 502 852-5248, Fax: 502 852-5248; e-mail: m0qiu001@yahoo.com

Supplementary Figures

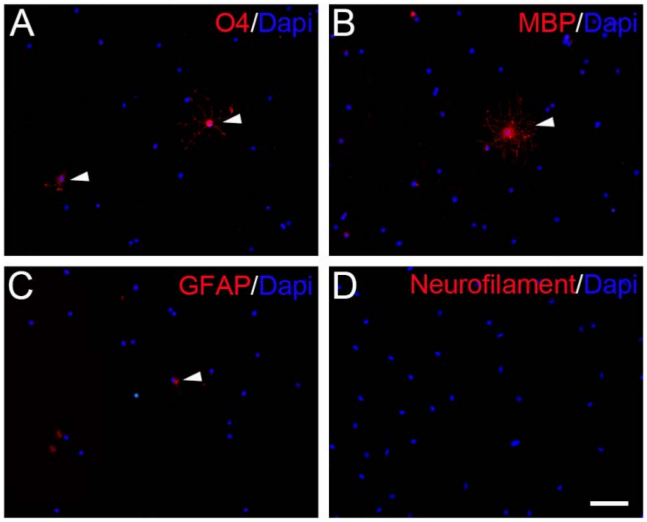

FIGURE S1. Immunostaining of primary mouse cortical OPCs with antibodies against O4, MBP, GFAP and Neurofilament. Positive cells were represented in arrows. Scale bars: 100 µm.

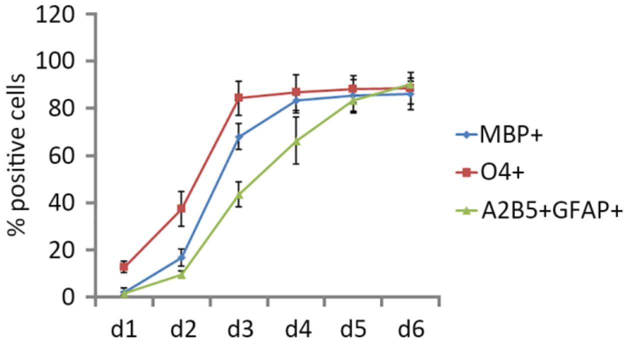

FIGURE S2. Quantification of O4+ and MBP+ cells during oligodendrocyte differentiation, and GFAP+ cells during astrocyte differentiation at difference time points. Statistical analyses are presented as mean ± s.d, n=3.

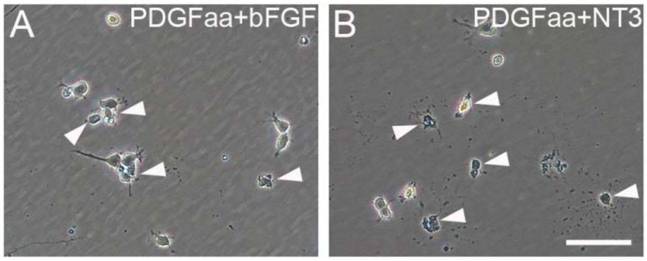

FIGURE S3. The morphology of primary mouse cortical OPCs after a 10-day culture in PDGFAA + bFGF (A) or PDGFAA + NT3 (B). Mouse OPCs could not maintain self-renewal and proliferation for a long time, cell divisions slowed down and eventually ceased followed by apoptosis (white arrows). Scale bars: 50 µm.

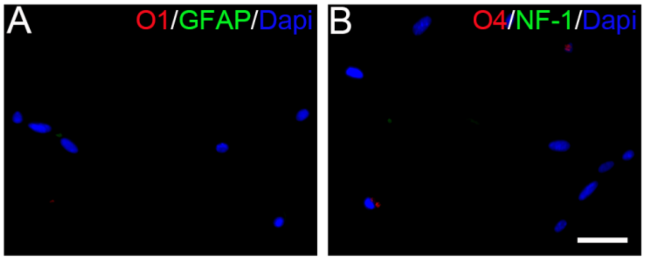

FIGURE S4. Lack of mature glial and neuronal marker expression in OPC-derived flat cells. The flat cells were induced from mouse primary OPC culture by the stimulation of EGF + bFGF + PDGFaa and then immunostained with anti-O1, anti-O4, anti-GFAP and anti-neurofilament (NF-1). Scale bars: 50 µm.
